# The role of NOD-like receptors in innate immunity

**DOI:** 10.3389/fimmu.2023.1122586

**Published:** 2023-03-15

**Authors:** Cássio Luiz Coutinho Almeida-da-Silva, Luiz Eduardo Baggio Savio, Robson Coutinho-Silva, David M. Ojcius

**Affiliations:** ^1^ Department of Biomedical Sciences, Arthur A. Dugoni School of Dentistry, University of the Pacific, San Francisco, CA, United States; ^2^ Laboratory of Immunophysiology, Biophysics Institute Carlos Chagas Filho, Federal University of Rio de Janeiro, Rio de Janeiro, Brazil

**Keywords:** innate immunity, inflammation, nod-like receptors, toll-like receptors, inflammasome, pathogen recognition receptors, pathogen-associated molecular patterns

## Abstract

The innate immune system in vertebrates and invertebrates relies on conserved receptors and ligands, and pathways that can rapidly initiate the host response against microbial infection and other sources of stress and danger. Research into the family of NOD-like receptors (NLRs) has blossomed over the past two decades, with much being learned about the ligands and conditions that stimulate the NLRs and the outcomes of NLR activation in cells and animals. The NLRs play key roles in diverse functions, ranging from transcription of MHC molecules to initiation of inflammation. Some NLRs are activated directly by their ligands, while other ligands may have indirect effects on the NLRs. New findings in coming years will undoubtedly shed more light on molecular details involved in NLR activation, as well as the physiological and immunological outcomes of NLR ligation.

## 1 Introduction

The mammalian immunity consists of an innate immune system with ancient genome-encoded and highly conserved receptors and a more recently acquired adaptive immune system. The innate immune system provides the initial response against microbial invasion and other sources of danger. One of the goals of this first line of defense is to generate an immediate and non-specific response to infection and to maintain the pathogen under control. Moreover, the innate immune responses induce and shape the optimal adaptive immune responses and stimulate the generation of effector and memory T- and B-cell responses. Innate immune mechanisms are also responsible for recycling damaged cells and for initiating the process of tissue repair.

This rapid defense mechanism is dependent on the ability of the innate immune system to recognize quickly potentially harmful molecules by means of its pattern recognition receptors (PRRs), which recognize conserved damage-associated molecular patterns (DAMPs) and pathogen-associated molecular patterns (PAMPs) (1–3). The innate immune system expresses a broad range of PRRs, such as the family of Toll-like receptors (TLRs), retinoic acid-inducible gene-I (RIG-I)-like receptors (RLRs), C-type lectin receptors (CLRs), and the nucleotide-binding domain (NOD), leucine-rich repeat-containing protein receptors (NLRs) (4, 5).

This article will emphasize the role of NLRs in innate immunity.

## 2 NOD-like receptors

### 2.1 Introduction to NOD-like receptors

The NLR family of receptors is expressed primarily in the cytosol of immune cells, including macrophages, dendritic cells, and lymphocytes. However, NLRs are also expressed in some non-immune cells such as epithelial and mesothelial cells (3, 6).

The NLRs play a key role in sensing molecules associated with intracellular infection and stress. Thus, they sense a variety of generic stimuli that indicate intracellular microbial infection and damage, such as fluctuations in ion concentrations and generation of reactive oxygen species (ROS).

Three major signaling pathways can be activated downstream of NLRs: nuclear factor-κB (NF-κB) signaling, mitogen-associated protein kinase (MAPK) signaling, and inflammasome activation (6). Some NLRs are specialized to promote the activation of an intracellular complex called inflammasomes.

**Figure 1:**
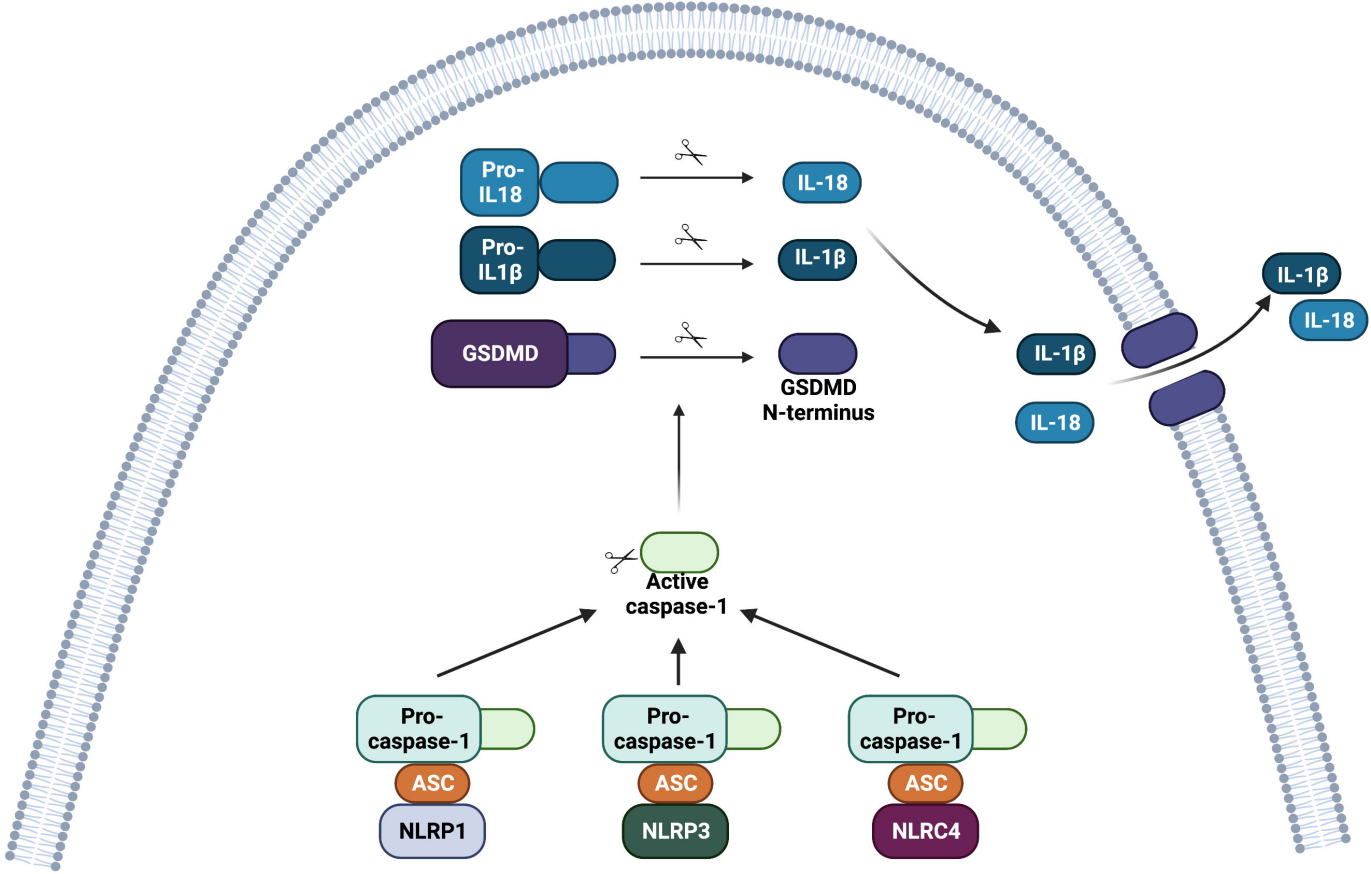
Structure of the inflammasome complexes involving NOD-like receptors (NLRs). After ligand recognition, NLRP1, NLRP3 and NLRC4 bind to pro-caspase-1 via the adaptor ASC. The CARD domain of ASC is necessary to recruit pro-caspase-1. Nlrp1b and NLRC4 might also directly bind to pro-caspase-1 since these receptors contain the domain CARD. The assembly of the inflammasome complex leads to its oligomerization. Activation of the inflammasome complex (NLRP1, NLRP3 or NLRC4) ultimately leads to proteolytic activation of pro-caspase-1 into active caspase-1. Caspase-1 then cleaves gasdermin D (GSDMD) into a C-terminal portion and an N-terminal portion. The N-terminal portion inserts into the plasma membrane and oligomerizes to form pores that can lead to a type of cell death called pyroptosis, and contribute to the release of inflammasome-related cytokines. The active form of caspase-1 also cleaves pro-IL-1β and pro-IL-18 into their active forms, IL-1β and IL-18, respectively. The active form of these pro-inflammatory cytokines is then released from the cell.

Inflammasomes were discovered in 2002 (7). They are cytoplasmic multi-protein complexes that assemble in the host cell in response to PAMPs or different types of stress (7, 8). Different inflammasomes formed by distinct NLRs converge on activation of pro-caspase-1 into its active caspase-1 form. Cleaved caspase-1, in turn, processes pro-interleukin (IL)-1β and pro-IL-18 into their active mature forms, IL-1β and IL-18. These cytokines can be released to the extracellular compartment in their active forms (**Figure 1**).

In parallel, active caspase-1 can cleave gasdermin D. The N-terminal fragment of gasdermin D mediates pyroptosis, which is a pro-inflammatory, lytic type of cell death that participates in the release of mature IL-1β and IL-18 from the cell (**Figure 1**) (9, 10). NLRP1, NLRP3, and NAIP-NLRC4 can activate the inflammasome, and each one of them will be discussed separately in this review.

The NLR gene family includes a protein that shares similarities with the NB-LRR subgroup of disease-resistance genes in plants, which highlights the extent of evolutionary conservation between these genes (5). Most of the known functions for NLRs are related to immunity and homeostasis (6, 11–13). Polymorphisms in several genes encoding the NLRs are associated with inflammatory and autoimmune diseases (6). However, the function of these receptors is not exclusively related to immunity, since they also play a role in diverse functions such as reproduction and embryonic development (14–19). In this review, we will focus on NLRs that play a role in innate immune responses.

### 2.2 Structure and classification of the NOD-like receptors

The NLR family of receptors is organized in a tripartite structure. The characteristic feature of NLR proteins is the presence of a central nucleotide-binding domain NACHT that orchestrates the self-oligomerization that occurs during activation; C-terminal leucine-rich repeats (LRRs) that are involved in sensing agonist or binding ligand; and a variable N-terminal homotypic protein-protein interaction domain, which is critical for downstream signaling (6, 11, 20). To date, in silico analysis has led to identification of 22 human and 34 NLR members (21–23).

The NLRs belong to five subfamilies (NLRA, NLRB, NLRC, NLRP, NLRX) depending on the structure of their N-terminal effector domains (6, 11). NLRs with an N-terminal portion consisting of an acidic transactivating domain are called NLRA (CIITA), which were previously known as transcriptional regulators of major histocompatibility complex (MHC) class II antigen presentation (24). The NLRB (NAIP) subfamily has an N-terminal domain containing baculoviral inhibition of apoptosis protein repeat (BIR)-like domain and is involved in host defense and cell survival (6, 11, 20). The N-terminal domain of the NLRC subfamily (NLRC1-5) contains a caspase activation and recruitment domain (CARD), which allows for direct interaction with other proteins carrying the CARD domain (11). The NLRP subfamily (NLRP1-14) expresses an N-terminal pyrin domain (PYD), and is involved in promoting the assembly and activation of the inflammasome (3, 11). The NLRX subfamily consists of only one member, NLRX1 (NOD9), which does not share significant homology with the N-terminal domain of the other four subsets. NLRX1 has an N-terminal sequence that targets the protein for migration to the outer mitochondrial membrane (6, 11).

**Table 1** summarizes the classification (NLR family), NLR genes present in humans and mice, their structural organization, and main functions. The symbols for the gene names were approved by the Human Genome Organization Gene Nomenclature Committee (HGNC) (5). This review will focus on the main NLRs belonging to each NLR family.

**Table 1:** Human and murine NOD-like receptors symbols, structure, their respective NLR family and main functions.

NLR family	HGNC-approved symbol		Structure (N-terminal –> C-terminal)	Main functions
	HUMAN	Mouse		
**NLRA**	*CIITA*	*CIIta*	CARD-AD-NACHT-NAD-LRR CARD-AD-NACHT-NAD-LRR	Regulation of MHC class II expression (25)
**NLRB**	*NAIP*	*Naip1* *Naip2* *Naip3* *Naip4* *Naip5* *Naip6* *Naip7*	BIRx3-NACHT-LRR BIRx3-NACHT-LRR BIRx3-NACHT-LRR BIRx3 BIR BIRx3-NACHT BIRx3-NACHT BIRx3-NACHT-LRR	Recognition of DAMPs and inflammasome formation associated with NLRC4 (26)
**NLRC**	*NOD1*	*Nod1*	CARD-NACHT-NAD-LRR CARD-NACHT-NAD-LRR	Induction of inflammation, disease, and response to microbial infection (11, 13, 27)
	*NOD2*	*Nod2*	CARDx2-NACHT-NAD-LRR CARDx2-NACHT-NAD-LRR	
	*NLRC3*	*Nlrc3*	CARD-NACHT-NAD-LRR CARD-NACHT-NAD-LRR	
	*NLRC4*	*Nlrc4*	CARD-NACHT-NAD-LRR CARD-NACHT-NAD-LRR	Formation of inflammasome associated with NAIP (13, 26)
	*NLRC5*	*Nlrc5*	CARD-NACHT-NAD-LRR CARD-NACHT-NAD-LRR	Regulation of MHC class I expression (28)
**NLRP**	*NLRP1*	*Nlrp1a* *Nlrp1b* *Nlrp1c*	PYD-NACHT-NAD-LRR-FIIND-CARD NACHT-NAD-LRR-FIIND-CARD NACHT-LRR-CARD NACHT-LRR	Formation of inflammasome and response to infection (13)
	*NLRP2*	*Nlrp2*	PYD-NACHT-NAD-LRR PYD-NACHT-NAD-LRR	Regulation of inflammation and reproduction (16, 29)
	*NLRP3*	*Nlrp3*	PYD-NACHT-NAD-LRR PYD-NACHT-NAD-LRR	Formation of inflammasome and response to infection (8)
	*NLRP4*	*Nlrp4a* *Nlrp4b* *Nlrp4c* *Nlrp4d* *Nlrp4e* *Nlrp4f* *Nlrp4g*	PYD-NACHT-NAD-LRR PYD-NACHT-NAD-LRR PYD-NACHT-NAD-LRR PYD-NACHT-NAD-LRR PYD-NACHT-NAD-LRR PYD-NACHT-NAD-LRR PYD-NACHT-NAD-LRR PYD-NACHT-NAD-LRR	Regulation of inflammation (30)
	*NLRP5*	*Nlrp5*	PYD-NACHT-NAD-LRR NACHT-NAD-LRR	Regulation of reproduction (16)
	*NLRP6*	*Nlrp6*	PYD-NACHT-NAD-LRR PYD-NACHT-NAD-LRR	Formation of inflammasome and response to infection (8)
	*NLRP7*	*-*	PYD-NACHT-NAD-LRR	Regulation of reproduction (17)
	*NLRP8*	*-*	PYD-NACHT-NAD-LRR	Possible role in inflammation (31, 32)
	*NLRP9*	*Nlrp9a* *Nlrp9b* *Nlrp9c*	PYD-NACHT-NAD-LRR PYD-NACHT-NAD-LRR PYD-NACHT-NAD-LRR PYD-NACHT-NAD-LRR	Regulation of inflammation (33, 34)
	*NLRP10*	*Nlrp10*	PYD-NACHT-NAD	Induction of inflammation, disease, and response to microbial infection (8)
	*NLRP11*	*-*	PYD-NACHT-NAD-LRR	Regulation of inflammation and NLRP3 inflammasome assembly and activation (35–37)
	*NLRP12*	*Nlrp12*	PYD-NACHT-NAD-LRR PYD-NACHT-NAD-LRR	Formation of inflammasome and response to infection (13)
	*NLRP13*	*-*	PYD-NACHT-NAD-LRR	Possible role in inflammation (31)
	*NLRP14*	*Nlrp14*	PYD-NACHT-NAD-LRR PYD-NACHT-NAD-LRR	Possible roles in inflammation and fertilization (18, 19, 38)
**NLRX**	*NLRX1*	*Nlrx1*	X-NACHT-NAD-LRR X-NACHT-NAD-LRR	Mostly unknown. Regulation of TLR-induced responses (39).

AD, acidic activation domain; CARD, caspase activating and recruitment domain; LRR, FIIND, domain with function to find; leucine-rich repeat; NACHT, domain present in CIITA, NAIP, HET-E, and TP-1; BIR, baculovirus inhibitor of apoptosis repeat; PYD, pyrin domain; and NAD, NACHT-associated domain; X, undefined. HGNC, Human Genome Organization Gene Nomenclature Committee

### 2.3 The role of NLRs in innate immunity

#### 2.3.1 CIITA/NLRC5

The Class II MHC transactivator (CIITA) is the sole member of the NLRA family, and has a characteristic acidic transactivating domain. CIITA was discovered in 1993 when it was shown to be defective in patients suffering from type II bare lymphocyte syndrome, which results in a complete loss of MHC class II expression in affected individuals (40). Mice lacking the *CIIta* gene show impairment of MHC class II expression that is tissue-specific, diminished T cell-dependent antigen responses, and decreased MHC class II-dependent allogeneic responses (41). Constitutive expression of CIITA is found in antigen-presenting cells (25), and exposure to IFN-γ can induce *de novo* expression of CIITA in most cell types (40).

MHC class II gene expression is regulated through conserved *cis-*acting promoter elements (W/S, X, X2, and Y), which interact with *trans*-activating DNA binding factors regulatory factor X (RFX), cAMP-responsive element binding protein (CREB), and nuclear factor Y (NF-Y) (25). The DNA binding factors are expressed constitutively, but their ligation to MHC class II genes is not sufficient to increase expression (25). CIITA does not bind to DNA directly. Instead, to induce MHC class II gene expression, CIITA associates with each of the DNA binding factors through protein-protein interactions, forming a transcriptionally active complex, or “enhanceosome” (25, 42). Therefore, the main function of CIITA appears to be the regulation of antigen presentation.

Cell-type specific promoters (PI, PIII, and PIV) regulate the expression of CIITA in different cell types (43). CIITA is expressed from one of these promoters which also encode a unique exon 1 that is spliced into a common exon 2 (43). PI is induced by macrophages and dendritic cells, PIII-mediated CIITA expression is constitutive in B cells, and PIV is induced in nonhematopoietic antigen presenting cells (APCs). A study published in 2018 demonstrated that the transcription factor NFAT5 is needed for the expression of CIIta and MHC class II in murine macrophages, but not in conventional myeloid dendritic cells (44). The authors demonstrated that NFAT5 regulates CIITA expression *via* a region distally upstream of *CIIta* (44). Future studies will define the regulation of CIITA in different cell types, including in dendritic cells in which the regulatory control of CIITA is less-well known.

In addition, CIITA is involved in basal transcription (25, 45, 46). It was reported that CIITA is an unusual serine-threonine kinase since it expresses both auto- and trans-phosphorylation activity, and CIITA phosphorylates the same targets as the TATA-box binding protein associated factor 1 (TAF1 - vital protein during transcription initiation) (45). Additionally, CIITA has been shown to have acetyltransferase activity, which bypasses a promotor requirement for TAF1 (46).

As reviewed elsewhere (47), several intracellular pathogens inhibit the expression of CIITA and, therefore, MHC class II molecules, to promote their survival. Conversely, CIITA also exhibits an antiviral restriction factor activity, which has been classified as intrinsic immunity. Intrinsic immunity refers to a type of innate immunity that can restrict viral replication directly, which can be induced by pre-existing factors in the host cell or factors that can be further induced by viral infection (48). Even though CIITA has restriction factor activities against HTLV-1 infection (49), CIITA was recently shown to inhibit infections by Ebola virus and coronaviruses, including SARS-CoV-2 (50). CIITA activates the expression of CD74 p41, which blocks cathepsin-mediated processing of the Ebola glycoprotein. Additionally, CD74 p41 blocks entry of coronaviruses, including SARS-CoV-2, *via* the endosomal pathway (50).

The CIITA protein is a coactivator for transcription of MHC class II genes. Additionally, CIITA enhances MHC class I gene expression (46) and regulates basal transcription initiation, but it also regulates transcription of over 60 immune genes, such as genes encoding for IL-4 and IL-10 (25, 45). These roles make CIITA an important regulator of innate and adaptive immunity.

Consistent with the distinctive immunomodulatory effects of CIITA, another NLR has been shown to regulate immune responses at the transcriptional level. More recently, the NLR family member, CARD domain containing 5 (NLRC5), was shown to regulate transcription of MHC class I genes (51–53).

Similarly to CIITA, NLRC5 can be induced by IFN-γ, and expression of NLRC5 results in increased MHC class I expression in epithelial and lymphoid cells (51). NLRC5 can induce expression of mediators of MHC class I antigen presentation, such as β2 microglobulin (β2M), transporter associated with antigen processing 1 (TAP1) and latent membrane protein 2 (LMP2) (28). More recent works describe the role of NLRC5 in pro-inflammatory responses, cancer, and inflammasome activation (53–55).

#### 2.3.2 NAIP/NLRC4

**Figure 2:**
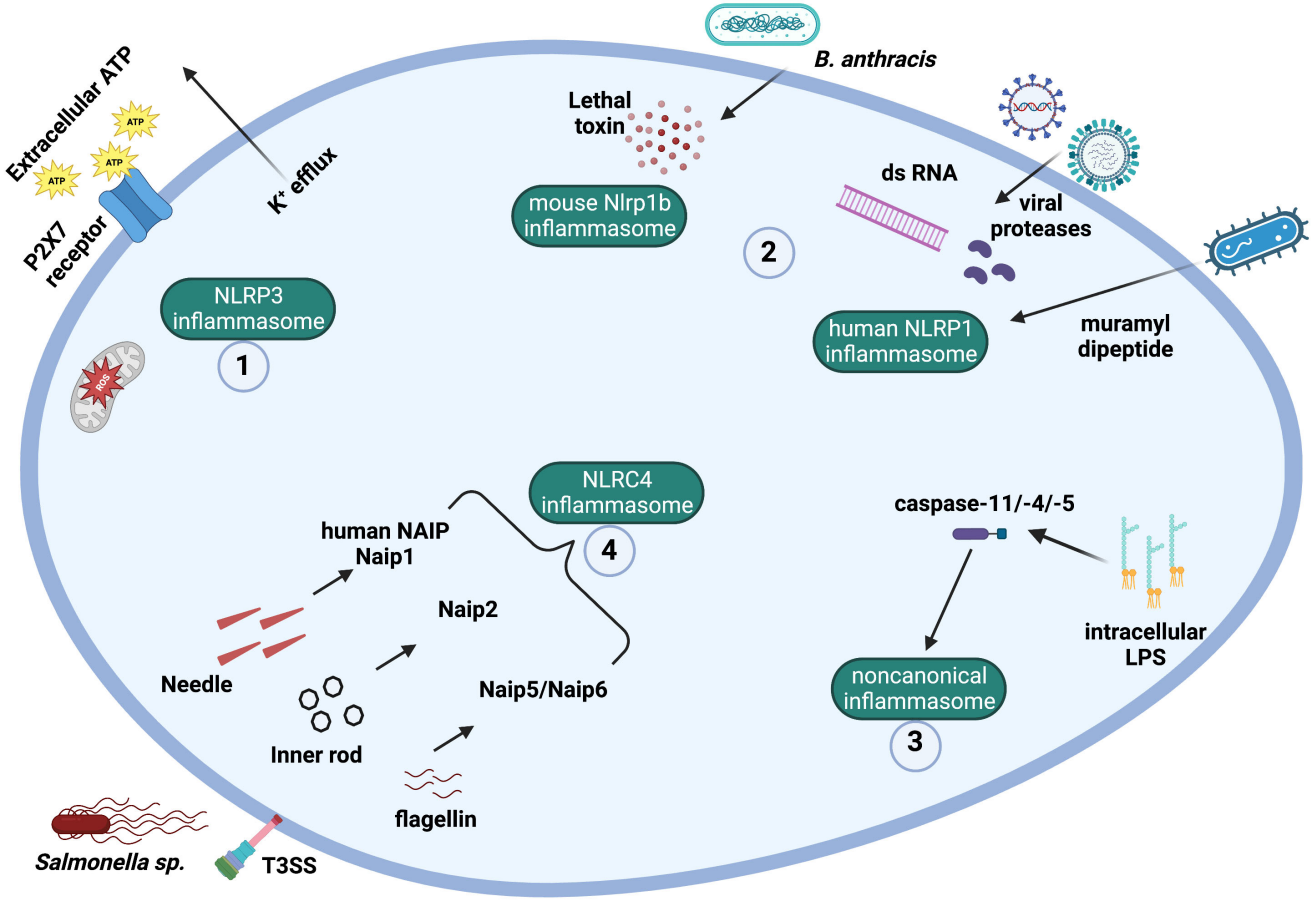
Mechanisms and ligands involved in canonical and noncanonical inflammasome activation. (1) The NLRP3 inflammasome can be activated by a plethora of signals, including potassium efflux, mitochondrial ROS production, and extracellular ATP ligation to P2X7 receptor. (2) Muramyl dipeptide, SARS-CoV-2 infection, viral proteases and double-stranded viral RNA can lead to the activation of the human NLPR1 inflammasome. The lethal toxin produced by *Bacillus anthracis* activates murine Nlrp1b. (3) The noncanonical inflammasome is activated by intracellular LPS from Gram-negative bacteria. Intracellular LPS directly binds to caspase-11 (in mice) or caspase-4/-5 (in humans), which become activated. (4) The protein NAIP in humans can recognize T3SS (type III secretion system) needle protein, while the different rodent Naips can recognize different bacterial ligands, such as T3SS needle protein (Naip1), T3SS inner rod protein (Naip2), and flagellin (Naip5 and Naip 6). Following PAMP ligation, NAIPs bind to and activate the NLRC4 inflammasome.

The NLR family apoptosis inhibitory protein (NAIP) belongs to the NLRB family. Initial studies on *NAIP* suggested a correlation between internally deleted/mutated forms of *NAIP* transcripts and spinal muscular atrophy (SMA), while deletions/mutations in the *NAIP* transcript were not found in unaffected individuals (56). However, it was later suggested that a neighboring gene, *survival motor neuron* (*SMN*), was involved in SMA pathogenesis (57). A role for NAIP during SMA pathology remains to be completely understood. Nevertheless, NAIP is known to play a role in inflammasome formation (**Figure 2**) (58–60), and apoptosis inhibition (61–63).

Humans express a single functional copy of the *NAIP* gene, while rodents express several *Naips* (seven intact copies in the 129 murine strain) (5, 64). In mice, each NAIP recognizes a single bacterial ligand from either the T3SS (type III secretion system) or bacterial flagella. Naip1 responds to the T3SS needle protein and Naip2 to the T3SS inner rod protein, while Naip5 and Naip6 respond to flagellin. However, human NAIP responds only to the T3SS needle protein and does not recognize flagellin (**Figure 2**) (26).

After recognition of the PAMP, some NAIPs bind the NLR family CARD domain containing 4 (NLRC4) protein, which then oligomerizes. The NLRC4 CARD domain activates caspase-1 (26, 59) (**Figures 1**, **2**). Among inflammasomes, only NLRC4 is downstream from the upstream NAIP (12, 26, 59). In the NAIP-NLRC4 inflammasome, NAIP molecules recognize the pathogen (or PAMPs) and assemble with NLRC4 molecules, which recruit and activate caspase-1 (26). The NAIP-NLRC4 inflammasome confers protection against infections with *Legionella pneumophila*, *Salmonella thyphimurium*, and *Shigella flexneri* (58, 60). Additionally, the NLRC4 inflammasome (along with the NLRP3 inflammasome) were involved in immune responses against *Candida albicans* in a murine model of infection (65).

It was reported that NLRC4-deficient murine macrophages challenged with *S. thyphimurium* (the causative agent of salmonellosis) failed to activate inflammasomes (66, 67). On the other hand, NLRC4-overexpressing macrophages died when they were infected with high loads of *S. thyphimurium* (66, 68). These results suggest that the NLRC4 inflammasome may play a different role depending on the bacterial load. These data also suggest that moderate inflammatory responses may favor microbial clearance, while exacerbated inflammation induces host cell death and may be deleterious to the host.

The majority of studies on inflammasome activation were performed with macrophages. Interestingly, unlike macrophages, neutrophils infected with *S. thyphimurium* activate the NLRC4 inflammasome, but the infection does not induce inflammasome-dependent pyroptosis (69). Therefore, neutrophils can activate and sustain an NLRC4-dependent inflammasome response against *S. thyphimurium* infection. Because neutrophils do not die by pyroptosis after NLRC4 inflammasome activation, it is believed that these cells can maximize the host’s pro-inflammatory response against bacterial infection (66).

NAIP proteins are also involved in inhibition of apoptosis. NAIP and its BIRx3 domain inhibit cleavage of pro-caspase-3 by caspase-9 (which is activated by the apoptosome), thus preventing apoptosis at the initiation stage of apoptosome formation (61, 63). Thus, NAIP is a critical inhibitor of programmed cell death in host cells, and NAIP assembles with NLRC4 to form a fully functional inflammasome, which can lead to secretion of cytokines, induction of cell death, inflammation, and bacterial infection control.

#### 2.3.3 NOD1 and NOD2

Nucleotide-binding oligomerization domain containing 1 (NOD1) and nucleotide-binding oligomerization domain containing 2 (NOD2) were the first NLR members to be described. NOD1 and NOD2 contain one and two CARD domains, respectively (see **Table 1**). Since their discovery, NOD1 and NOD2 have been studied for their roles during microbial infection, inflammation, and disease.

Both NOD1 and NOD2 recognize peptidoglycan moieties (6). Both Gram-positive and Gram-negative bacteria express peptidoglycan, but they produce different motifs that can be sensed by either NOD1 or NOD2 (4). Thus, NOD1 responds to γ-D-glutamyl-mesodiaminopimelic acid (iE-DAP), which is produced by Gram-negative bacteria and some Gram-positive bacteria (70). NOD2 is a general sensor for muramyl dipeptide (MDP) motifs made by both Gram-negative and Gram-positive bacteria (71, 72), as well as single-stranded RNA from viruses (71, 73). NOD1 and NOD2 are expressed in a range of cells, from macrophages, dendritic cells, and keratinocytes, to endothelial cells and intestinal and lung epithelial cells (4).

Following detection of their ligands, NOD1 and NOD2 oligomerize and recruit receptor-interacting serine-threonine kinase 2 (RIPK2, also called RIP2 or RICK) *via* their CARD domain (74). RIPK2 leads further to the recruitment of X-linked inhibitor of apoptosis protein (XIAP), IAP1, cIAP2, and TRAF2, TRAF5 and/or TRAF6 (TNF-receptor associated factors). RIPK2 is ubiquitinated and recruits transforming growth factor β-activated kinase 1 (TAK1), and TAB1, TAB2, and TAB3. This series of events lead to assembly of a multi-protein platform called the “nodosome”, which can lead to the activation of downstream signaling pathways, such as NF-κB and MAPK (74). Besides being involved in the NF-κB and MAPK signaling pathways, NOD1 and NOD2 play a role in the interferon regulatory factor (IRF) pathway and type I IFN responses (6). As expected, the NOD1 and NOD2 signaling pathways are tightly controlled (74).

The role of NOD1 and NOD2 in the host anti-microbial responses has been extensively studied *in vitro*, *in vivo* in mouse models, and in genetic susceptibility studies in humans (71, 74). NOD-induced immune responses are important against a wide variety of bacterial infections, such as infections with *Chlamydia trachomatis* (75), *Clostridium difficile* (76), *Escherichia coli* (77), *Legionella pneumophila* (78), *Listeria monocytogenes* (79), *Mycobacterium tuberculosis* (80), and many others. With regards to viral infections, NOD1 plays a role in the immune response against hepatitis C virus (81), and NOD2 regulates the IFN type I-anti-viral responses against the respiratory syncytial virus (73). Furthermore, NOD1 and NOD2 play a role in stimulating Th1 cell responses required for parasitic clearance in mouse models of infection with *Toxoplasma gondii* (82), *Trypanosoma cruzi* (83), and *Leishmania infantum* (84). NOD1 and NOD2 receptors are involved in the clearance and induction of immune responses required to eliminate these pathogens. However, less is known about the mechanism of recognition of parasitic infection: whether NOD1 and/or NOD2 directly recognize PAMPs from the parasites, or if the infection is recognized indirectly.

Some studies support the role of NODs in metabolic diseases through a mechanism of aberrant expression and activation of *NOD1* and *NOD2* genes. In fact, NOD1 and NOD2 are upregulated in monocytes from patients with type 2 diabetes, compared with healthy individuals (85). NOD1 expression is also upregulated in adipose tissues of women with gestational diabetes (86), and in the subcutaneous tissue from patients with metabolic syndrome (87).

Genetic variations of *NOD1* and *NOD2* have also been linked with inflammatory diseases. Loss-of-function polymorphisms in *NOD1* can lead to asthma and sarcoidosis, and *NOD2* mutations have been linked with Crohn’s disease and ulcerative colitis (6). Conversely, *NOD2* gain-of-function mutations correlate with autoinflammatory diseases, such as Blau syndrome/early-onset sarcoidosis in the skin, eyes, and joints (6). Overall, NOD1 and NOD2 signaling is important during host defense against microbes, and is associated with metabolic, autoimmune, and inflammatory diseases.

#### 2.3.4 NLRP1

NLR family pyrin domain-containing 1 (NLRP1) was the first NLR member discovered to participate in inflammasome and caspase-1 activation (7). Humans show a single functional copy of the *NLRP1* gene, while rodents express several paralogues of the same receptor, called *Nlrp1a, Nlrp1b,* and *Nlrp1c* (see **Table 1**).

The activation of human and murine NLRP1 leads to inflammasome activation, which can induce caspase-1 activation, processing and release of IL-1β and IL-18, and pyroptosis (**Figure 1**). Recently, Huang et al. (88) and Hollingsworth et al. (89) described the molecular mechanisms of NLRP1 activation.

NLRP1 is unique among the inflammasome-forming NLRs because it contains a C-terminal extension with a FIIND and a CARD domain (**Table 1**). Because the activation of the inflammasome can lead to cell death, this is a well-regulated process in the cell. The studies show that, in resting cells, the dipeptidyl peptidase DPP9 interacts with the FIIND domain of rat NLRP1 (88) and human NLRP1 (89) to suppress spontaneous NLRP1 activation. This mechanism may explain why the NLRP1 inflammasome is activated by stimuli that lead to degradation of the N-terminal domain of NLRP1 (90): the cleavage of the NLRP1 N-terminal domain by pathogen proteases leads to its proteasomal degradation, which frees the C-terminal domains involved in NLRP1 inflammasome assembly.

MDP activates the human NLRP1, while the virulence factor lethal toxin (LT), which is produced by the pathogen *Bacillus anthracis*, activates the murine Nlrp1b (**Figure 2**) (13, 91). This was the first example of an NLR that detects a virulence factor instead of a structural microbial element (6). More recently, diverse viral proteases (13, 91, 92) and double-stranded RNA (93) were shown to activate the NLRP1 inflammasome. SARS-CoV-2 infection was also demonstrated to activate the human NLRP1 inflammasome in human lung epithelial cells (**Figure 2**) (94). In this study, human NLRP1 was cleaved by multiple coronavirus proteases, which trigger inflammasome activation and death of the infected cell, thus limiting the generation of more virions (94). Further studies are needed to fully characterize the outcome of NLRP1 inflammasome activation during viral infection (95).

The NLRP1 inflammasome response has been shown to be essential against bacterial and parasitic infection (13, 91, 96). Interestingly, NRLP1 in rats and mouse confers resistance to *T. gondii* infection and is involved in the development of a complete immune response and parasite control (96).

Variations in NLRP1 have also been linked to autoimmune diseases (13). Defects in the NLRP1 gene increase the risk of developing several diseases, such as vitiligo (97), congenital toxoplasmosis (98), autoimmune thyroid disorders (99), systemic lupus erythematosus (100), Alzheimer’s disease (101), and rheumatoid arthritis (102).

#### 2.3.5 NLRP3

NLR family pyrin domain-containing 3 (NLRP3) plays a role primarily in the formation of an inflammasome complex. The NLRP3 inflammasome is the best characterized inflammasome. It had attracted early attention since it was demonstrated that an autosomal gain-of-function mutation in the *NLRP3* gene was linked to an inherited autoinflammatory disease called cryopyrin-associated periodic syndrome (CAPS) (103).

In fact, the study by Nakamura et al. showed that, during CAPS, mast cells in the skin were the main cell population responsible for inducing urticarial rashes (104). The study demonstrated that, unlike normal mast cells which require stimulation for IL-1β secretion, mast cells from CAPS patients constitutively produced IL-1β. The mast cell-derived IL-1β from CAPS patients induced recruitment of neutrophils and vascular leakage, histological landmarks of urticarial rash. These results explain why anti-IL-1β treatment alleviates the urticarial rash symptom in CAPS patients (104). A more recent study in mice reported that gain-in-function mutations in *Nlrp3* restricted to neutrophils, and to a lesser extent to macrophages and dendritic cells but not mast cells, are sufficient to induce severe CAPS (105). This recent study also shows that skin-infiltrating neutrophils are an important source of IL-1β. Together, these two studies demonstrate that mast cells and neutrophils can activate the NLRP3 inflammasome and play a crucial role in the pathophysiology of CAPS.

NLRP3 inflammasome activation requires two signals: first, a priming signal, which is provided by PAMPs such as LPS, leads to activation of the NF-κB pathway and consequent upregulation of NLRP3 and pro-IL-1β and pro-IL-18; and second, an activation signal, which is provided by a variety of stimuli, such as DAMPs. A long list of stimuli can activate the NLRP3 inflammasome, such as extracellular ATP, ROS generation and mitochondrial dysfunction, lysosomal damage and cathepsin B release from lysosomes, the ionophore nigericin, viral RNA, double-stranded RNA, uric acid crystals, asbestos crystals, silica, amyloid-β, K^+^ efflux, poly(I-C) acid, imidazoquinoline, and chitosan (8, 106, 107). NLRP3 is not thought to bind directly to its ligands since the NLRP3 inflammasome is activated by a wide range of stimuli that have little in common chemically or structurally (107). Therefore, it has been proposed that the NLRP3 inflammasome may sense the cellular stress induced by the different stimuli (**Figure 2**).

The structural domains contained in the NLRP3 protein are described in **Table 1**. The ultrastructure of the NLRP3 inflammasome has been recently described using cryogenic electron microscopy. It was shown that the NLRP3 inflammasome forms a disk-shaped structure, with the centrosomal kinase NEK7, and the adaptor protein ASC, which recruits the protease caspase-1 (108). For more details and an image of the NLRP3 assembly complex, we refer the readers to the recent article by Xiao et al. (108).

The NLRP3 inflammasome is essential for host immune responses against bacteria, fungi, and viral infections (107). In fact, it was demonstrated that the NLRP3 inflammasome is activated in human macrophages by SARS-CoV-2, leading to secretion of IL-1β and IL-18, and pyroptosis, which contributes to the hyperinflammatory state of the patient (109). Inhibition of NLRP3 inflammasome activation during SARS-CoV-2 infection was shown to reverse chronic lung pathology (109). In addition, the NLRP3 inflammasome is involved in the development of metabolic disorders such as atherosclerosis (110), gout (111), and type 2 diabetes (112); autoimmune diseases such as rheumatoid arthritis (113); and diseases of the central nervous system such as Alzheimer’s disease (114).

The NLRP3 inflammasome activation pathway following the two-signal model described above is known as canonical NLRP3 inflammasome activation, which leads to activation of caspase-1, resulting in IL-1β/IL-18 secretion and pyroptosis (**Figure 1**). Additionally, a non-canonical NLRP3 inflammasome activation has been described, which involves caspase-11 (in mice) or caspase-4/-5 (in humans) (**Figure 2**) (115). The non-canonical NLRP3 inflammasome activation is initiated by direct detection of cytosolic LPS by caspase-11/-4/-5, leading to pyroptosis similarly to that induced by caspase-1 (116). However, the secretion of mature IL-1β and IL-18 after caspase-11/-4/-5 activation is caspase-1-dependent (115, 116).

More recently, NLRP11 has been proposed to be involved in NLRP3 inflammasome assembly and activation (35). It was demonstrated that human NLRP11 binds to ASC by PYRIN domains and binds to NLRP3 *via* its NACHT-LRR region. NLRP11 acts as a scaffold that connects ASC and NLRP3 (35). The specific deletion of human NLRP11 in macrophages prevented NLRP3 inflammasome activation, polymerization of NLRP3 and ASC, caspase-1 activation, pyroptosis and cytokine release. However, NLRP11 had no effect on other inflammasomes (35). NLRP11 is present in humans but is not expressed in mice. Future studies are needed to confirm and elucidate the role of NLRP11 in NLRP3 inflammasome assembly and activation.

In clinical settings, the NLRP3 inflammasome is being considered as a therapeutic target given its role in several diseases. Inhibitors with therapeutic potential targeting NLRP3 pathways have already been described. They are commercially available for research purposes (117), such as MCC950, which binds directly to the NLRP3 molecules, blocking their ATPase domains and resulting in NLRP3 inhibition.

#### 2.3.6 NLRX1

NLR family X1 (NLRX1), the sole member of the NLRX family, has an atypical N-terminal X domain and lacks obvious homology with other NLRs (6, 11). Its N-terminal domain presents a mitochondria-signaling sequence that sorts NLRX1 molecules to mitochondria. This NLR is ubiquitously expressed in mammalian tissues, with the highest expression levels being in cells of the heart, muscles, and mammary glands (118).

To date, NLRX1 has been reported to dampen the TLR-induced NF-κB (119) and type-I interferon signaling pathways (120), regulate ROS production (121–123), induce autophagy (5), regulate cell death (25, 40–42), and modulate c-Jun N-terminal kinase (JNK) and MAPK signaling pathways (39). On the other hand, other studies report that NLRX1 amplifies NF-κB responses by inducing ROS production (121, 123), and that NLRX1 enhances ROS production, which favors chlamydial infection (122).

Unlike other NLR proteins, NLRX1 does not form inflammasome complexes. Instead, it acts as a negative regulator of inflammatory responses. However, the set of PAMPs and DAMPs sensed by this receptor and its functions and mechanisms of action are not fully understood.

## 3 Concluding statement

PRRs did not attract much interest in the immunological community until Toll in *Drosophila* and TLRs in mammals were shown to play a critical role in the initiation of the innate immune response (124). Since then, more research has been conducted and the NLRs were discovered to be involved in a wide range of physiological mechanisms, including immunity regulation, responses to microbial infections, fertilization, and reproduction. Research on the functions of the NLRs was begun more recently, but progress has been made quickly to identify ligands and downstream pathways. Research in this field is rapidly uncovering mechanistic details about steps that initiate NLR-dependent signaling pathways, and their role in physiology and immunology *in vivo*. Future research should uncover new roles for NLRs and help us to better understand the molecular mechanisms involved in their activation and role in immunity and disease.

NLRP3 is involved in formation of the most studied inflammasome to date, the NLRP3 inflammasome. Even though a long list of stimuli have been demonstrated to activate this inflammasome, it is unlikely that NLRP3 directly binds to each of the ligands. It remains to be completely determined how such different stimuli can activate the same receptor to activate the NLRP3 inflammasome. A possibility is that the NLRP3 inflammasome senses global cellular stress by means of the different stimuli.

Understanding the mechanisms of activation of NLRs is clinically significant, as the research may suggest potential therapeutic targets in several pathological conditions. In fact, NLRP3 inhibitors have already been considered in clinical trials involving diseases modulated by the NLRP3 inflammasome and IL-1β, such as in CAPS (125, 126) and in COVID-19 (127, 128). Future clinical trials and basic research will continue to unravel the potential to target inflammasome-associated NLRs for disease treatment and prevention.

## Author contributions

CA-d-S, LS, RC-S, and DO have contributed to writing and revising the text. All authors contributed to the article and approved the submitted version.
